# Immunotherapy change overcomes acquired resistance after chemo-immunotherapy in unresectable biliary tract cancer

**DOI:** 10.1007/s00262-025-04181-2

**Published:** 2025-09-29

**Authors:** Xiangqi Chen, Zixiang Zhou, Shuofeng Li, Guanhua Yu, Shi Feng, Ze Zhou, Boyu Sun, Shanshan Wang, Kai Liu, Chengjie Li, Mingjian Piao, Mingming Wang, Yihong Zhang, Hu Li, Chengpei Zhu, Zhenyu Zhu, Haitao Zhao

**Affiliations:** 1 Department of Liver Surgery, Peking, Union Medical College Hospital, Chinese Academy of Medical Sciences & Peking Union Medical College, Beijing, China; 2 Department of Hepatobiliary Surgery, The Fifth Medical Center of PLA General Hospital, Beijing, China; 3 Department of General Surgery Center, Clinical Center for Liver Cancer, Beijing Youan Hospital, Capital Medical University, Beijing, China; 4 Eight-year Medical Doctor Program, Chinese Academy of Medical Sciences & Peking Union Medical College, Beijing, China

**Keywords:** Biliary tract cancer, Combination therapy, Immunotherapy, Drug resistance

## Abstract

**Purpose:**

To assess the incidence, clinical characteristics, and post-progression management strategy of resistance to immunotherapy–chemotherapy combination in unresectable biliary tract carcinoma (uBTC).

**Experimental:**

**Design:**

Patients with uBTC from multiple centers who received immunotherapy–chemotherapy combination were retrospectively included. Baseline characteristics, treatments, pattern of progression, and posttreatment managements were recorded. The primary endpoint was post-progression survival (PPS).

**Results:**

Out of 194 patients, 130 (67.0%) developed resistance, including 78 (40.2%) with acquired resistance (AR) and 52 (26.8%) with primary resistance (PR). Normal CA19-9 level and combining target therapy were more common in patients with AR. Patients with both AR and PR commonly experience deterioration in general condition and systemic progression. Patients with AR and PR showed no difference in patterns of progression or current post-progression management strategies. A total of 103 (79.2%) patients receiving post-resistance antitumor treatments showed improved prognosis than those receiving best supportive care, while patients with AR received additional survival benefit than those with PR. Changing immunotherapy regimen after resistance brings significant survival benefit in patients with AR (mPPS: 15.1 vs. 9.50 months, HR = 0.41), but not those with PR. Adding/switching target therapy regimens (HR = 0.39) and local regional therapies (LRT) (HR = 0.36) after resistance brings potential benefit in patients with PR.

**Conclusions:**

Changing immunotherapy regimen is a promising strategy for overcoming AR to first-line immunotherapy–chemotherapy combination in uBTC, while adjusting targeted therapy and adding LRT may help overcome PR.

**Supplementary Information:**

The online version contains supplementary material available at 10.1007/s00262-025-04181-2.

## Introduction

Biliary tract cancer (BTC), encompassing intrahepatic cholangiocarcinoma (ICC), extrahepatic cholangiocarcinoma (ECC), gallbladder cancer (GBC), and other rarer subtypes, is a rare, heterogeneous, and highly aggressive malignancy^[[1]]^. Most of the BTC cases are diagnosed at an unresectable stage, and relies on systemic therapies^[[2]]^. Immune checkpoint inhibitor (ICI)–chemotherapy combinations have demonstrated promising clinical benefits in unresectable BTC (uBTC), and were recognized as first-line standard of care^[[3–5]]^. Despite such success, a large proportion of patients failed to achieve durable response to first-line ICI–chemotherapy combinations, and will ultimately develop resistance, resulting treatment failure. Resistance to ICI therapy is generally classified into two types: primary resistance (PR), in which patients exhibit no initial response and experience rapid disease progression, and acquired resistance (AR), in which patients initially respond to treatment but eventually develop disease progression^[[6,7]]^. AR is frequently observed in various types of tumors^[[8–10]]^, and is believed to have distinct underlying mechanisms and prognostic implications compared to PR^[[11,12]]^.

Current second-line therapy for BTC relies heavily on chemotherapy regimens, and brings limited survival benefit ^[[13,14]]^. Attempts have been made to establish new therapeutics such as target therapies, immunotherapies, and local regional therapies (LRT) in the post-progression treatments of advanced BTC, but most of them remain in an early stage and lack solid evidence^[[15–17]]^. Response and resistance to previous line of treatment may provide crucial clue for post-progression management strategies. Hence, we conducted a multicenter retrospective cohort study, aiming to assess the incidence, clinical characteristics, and prognostic factors associated with resistance in patients with uBTC receiving ICI–chemotherapy combination, and to explore an individualized post-progression management strategy based on characteristics of resistance.

## Methods

### Patient collection and response assessment

This retrospective study included patients with unresectable BTC (uBTC) receiving treatment with ICI combined with chemotherapy at Peking Union Medical College Hospital (PUMCH) and the Fifth Medical Center of the PLA General Hospital (PLAGH) from 2018–05 to 2024–10. Patients were eligible if they met the following criteria: (1) Their age was between 18 and 80 years; (2) They were histologically confirmed BTC; (2) They exhibited at least one measurable lesion according to the Response Evaluation Criteria in Solid Tumors (RECIST) version 1.1; (4) They were not eligible for curative surgical treatment; (5) Their Child–Pugh class was A–B; (6) Their Eastern Cooperative Oncology Group (ECOG) performance status (PS) score was 0–2. Patients with end-stage BTC, history of organ transplantation, prior systemic treatment, discontinued use of combination therapy after less than two cycles of treatment, more than one type of tumor, or an unclear or controversial diagnosis, were excluded. Patient collection and identification of duplicated cases was done by researchers not participated in patient treatment. The study was conducted in accordance with the principles of the Declaration of Helsinki^[[18]]^ and was approved by the Institutional Review Board of PUMCH (No. I-24PJ2728, I-24PJ2730, and I-25PJ0158) and PLAGH (No. KY-2025–1-18–1). All patients provided written informed consent before receiving ICI–chemotherapy combination and relevant target therapies. All patients underwent first-line ICI combined with gemcitabine-based chemotherapy. The choice of systemic regimen was determined based on patient preference after a comprehensive discussion of the latest efficacy and safety data, treatment cycles, and cost. ICI was administered every three weeks at varying doses: pembrolizumab^[[19]]^, camrelizumab^[[20]]^, sintilimab^[[21]]^, tislelizumab^[[22]]^ (200 mg), and toripalimab^[[23]]^ (240 mg), or devalumab^[[3]]^ (1500 mg). The chemotherapeutic regimens consisted of gemcitabine plus oxaliplatin (GEMOX) ^[[24]]^ or cisplatin (GC) ^[[4]]^ and dynamically adjusted based on the patients’ adverse events. The combination of lenvatinib was decided according to treating physicians’ judgment and patient’s willingness. Lenvatinib was orally administered once daily (8 mg/day for body weight < 60 kg or 12 mg/day for body weight ≥ 60 kg) ^[[24]]^. All patient characteristics at the start of treatment were collected from the outpatient medical records. Baseline variables considered in the analysis were as follows: age, sex, HBV infection, ECOG-PS, Child–Pugh subgroup, primary tumor location, tumor differentiation, tumor status, and CA19-9 level.

Response assessments were conducted every four to six weeks until the end of treatment. Treatment responses were determined according to RECIST ver. 1.1, consistent with the practice adopted in phase III clinical trials of chemo-immunotherapy^[[3,4]]^. All treatment response assessments were confirmed by trained radiologists based on computed tomography or magnetic resonance imaging results. Confirmatory imaging was required only for confirming complete/partial response when applicable. Confirmatory scans were not mandated for progression, and iRECIST was not initially applied to distinguish pseudoprogression. The treatment was continued until disease progression, death, or the occurrence of unacceptable adverse events. The disease was considered to have progressed when either radiological or clinical progression occurred. Radiological progression was defined as an increase in the size of existing tumors of more than 20% or the appearance of new lesions. Clinical progression was considered as continuous rise in CA19-9 level during two response assessments. AR was defined as disease progression after achieving a complete response (CR), partial response, or stable disease (SD) with a duration of 6 months or more ^[[7]]^. PR was defined as progressive disease (PD) as the best response or SD with a duration less than six months. Patients died or quitted without confirmed progression were not considered as resistance.

### Characteristics at resistance

Patients’ clinical characteristics were recollected at the time when patients were determined to have disease progression. The clinical characteristics recollected include tumor status, Child–Pugh subgroup, CA19-9 level, and ECOG-PS.

The pattern of progression was collected at the time of disease progression from the outpatient medical record. The pattern of progression was analyzed based on resistance subgroups and predefined characteristics: Oligoprogression and systemic progression were defined as progression in less than two or more than three disease lesions, respectively. Single- and multi-site progression were defined as progression in single or more than two organs and/or lymph node groups. Occurrence of new lesions was restricted to measurable lesions according to RECIST ver. 1.1.

### Post-resistance managements and follow-up

Patients were followed up after resistance to first-line combination therapy until death or loss to follow-up. Subsequent post-resistance managements were depended on a joint decision of treating physicians and patients. Data on post-resistance second-line management (including that performed at initial and other medical centers) were collected from outpatient medical records, while follow-up to survival was done by phone contact to patients and/or their close family members. Further lines of treatments after second-line treatment failure were not included. Only follow-up data up to 2025–04-30 were included in the analysis.

Subsequent post-resistance immunotherapies were analyzed based on resistance subgroups and immunotherapy strategies. Post-resistance immunotherapy strategies include: 1: Switching into new ICI regimen, 2: Continuing or stopping index therapy. Propensity score matching analysis was used to reduce post-resistance managements’ selection bias. Patients who switched into new ICI regimen were matched with those who continued or stopped index therapy using the propensity matched method as described by Rubin and Rosenbaum. The propensity score was estimated using a multivariable logistic regression model by including all clinical characteristics upon progression including age, sex, primary tumor location, type of resistance, ECOG-PS upon progression, Child–Pugh subgroup upon progression, tumor status upon progression, CA19-9 level upon progression, occurrence of new lesion, number of progressed lesions, and number of progressed sites. Balanced cohorts were created using 1-to-2 nearest-neighbor propensity score matching without replacement (caliper width 0.2 standard deviations).

Post-resistance response assessments were retrospectively reevaluated in patients who continued initial combination therapy after progression in order to identify any possible pseudoprogression. The patients’ post-resistance management must fulfill the following criteria: no change of immunotherapy regimen; no application of LRT; and continuation or withdrawal of the initial target and chemotherapy regimens. The reevaluation of treatment responses was based on the iRECIST criteria^[[25]]^. Pseudoprogression was defined as having iUPD (unconfirmed progression) and subsequent confirmation as iSD (stable disease), iPR (partial response), or iCR (complete response).

### Statistical analysis

Progression-free survival (PFS) was defined as the time from the first dose of ICI to radiological or clinical progression. Patients who died before any recognizable disease progression were excluded. Overall survival (OS) was defined as the time from the first dose of ICI to death from any cause. Post-progression survival (PPS) was defined as the time from radiological or clinical progression to death from any cause. The primary endpoint of this research was PPS. The second endpoint was OS. The categorical baseline characteristics were compared using a χ^2^ or Fisher’s exact test when appropriate, whereas the continuous characteristics were compared using Mann–Whitney U test. OS was calculated using a Kaplan–Meier survival analysis. Univariate and multivariate Cox regression analyses were used to identify risk factors for survival. All potential risk factors (*P* < 0.1) were included in the multivariate Cox regression model. Multicollinearity diagnostics were performed to exclude any relevant variants before multivariate Cox regression analysis. *P* < 0.05 was considered statistically significant. Statistical analyses were performed using SPSS Statistics (version 26.0; SPSS Inc.) and R statistical software (version 4.3.1; R Project for Statistical Computing).

## Results

### Patient cohort for resistance to ICI–chemotherapy combination

**Fig. 1:**
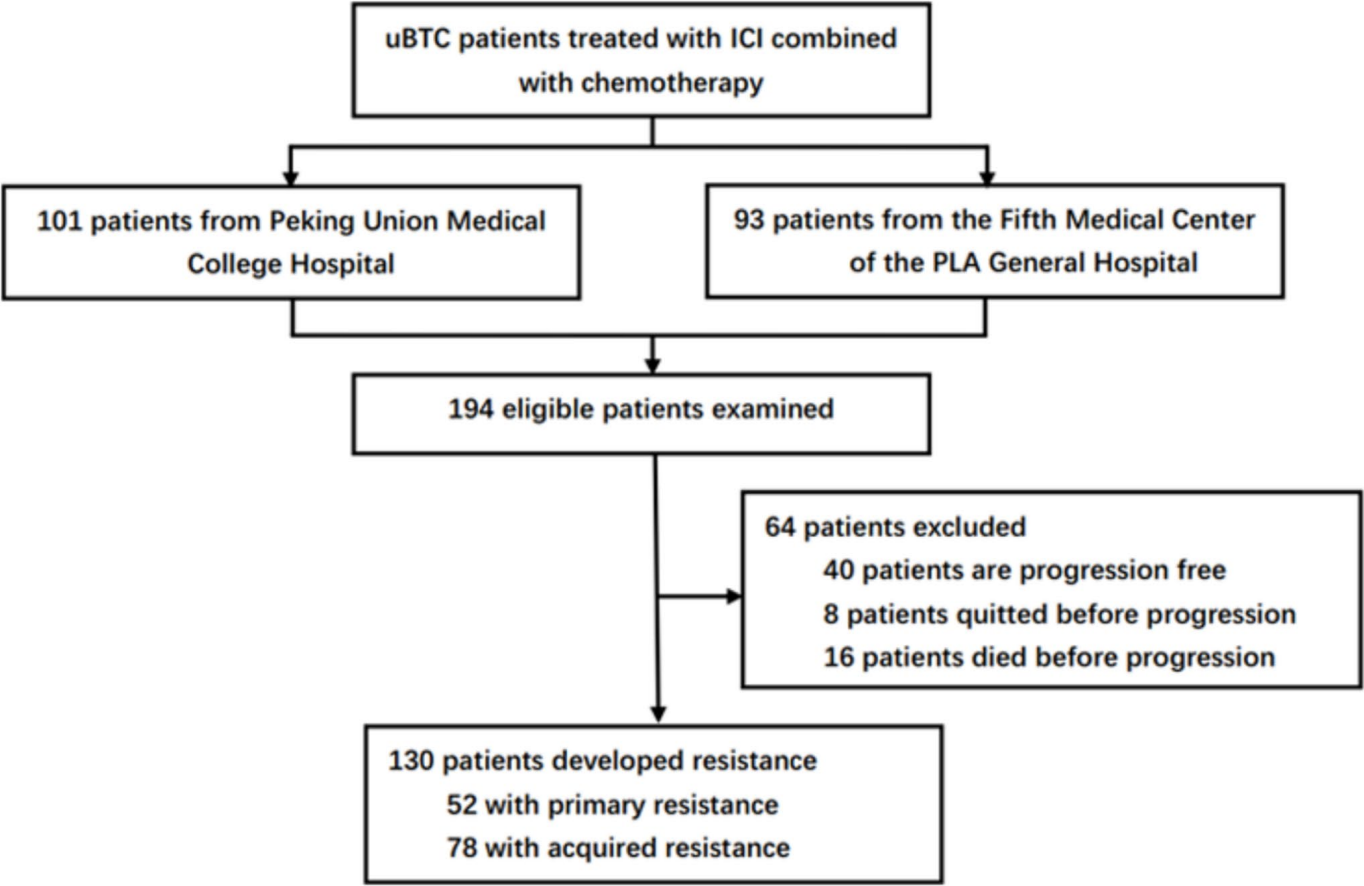
Patient enrollment flowchart of the cohort. uBTC, unresectable biliary tract cancer; ICI, immune checkpoint inhibitor;

A total of 194 patients with uBTC who received ICI combined with chemotherapy at PUMCH and PLAGH from 2018–05 to 2024–10 were analyzed, no duplicated cases were found. Of these, 40 patients who remained progression-free as of April 30, 2025, 8 who were quitted before progression, and 16 who died without documented progression were excluded. Ultimately, 130 patients were identified as resistant, including 78 with AR and 52 with PR. Baseline characteristics and treatment regimens of the study cohort are presented in Fig. 1 and Table 1

**Table 1:** Baseline characteristics of patients developing resistance

Characteristics	Patients (n, %)
Number (n)	130
PUMCH	68 (52.3)
PLAGH	62 (47.7)
Age median (IQR), years	58 [50–64.25]
*Sex*	
Male	90 (69.2)
Female	40 (30.8)
*HBV Infection*	
Yes	30 (23.1)
No	100 (76.9)
Tumor type	
ICC	99 (76.2)
ECC	11 (8.5)
GBC	20 (15.4)
*Tumor status*	
Locally advanced	42 (32.3)
Metastatic	88 (67.7)
*ECOG-PS*	
0	102 (78.5)
1	26 (20.0)
2	2 (1.5)
*Child–Pugh subgroup*	
A	111 (85.4)
B	19 (14.6)
*CA19-9* > *37*	
Yes	81 (62.3)
No	36 (27.7)
Not available	13 (10.0)
*ICI*	
aPD-1	107 (82.3)
aPD-L1	23 (17.7)
*Target therapy*	
Lenvatinib	49 (37.7)
No	81 (62.3)

IQR, interquartile range; HBV, hepatitis B virus; ICC, intrahepatic cholangiocarcinoma, ECC, extrahepatic cholangiocarcinoma; GBC, gallbladder cancer; ECOG-PS, Eastern Cooperative Oncology Group Performance Status; CA19-9, carbohydrate antigen 19–9; aPD-1, anti-programmed cell death protein 1 antibody; aPD-L1, anti-programmed cell death ligand 1 antibody; ICI, immune checkpoint inhibitor;

The median follow-up duration was 22.7 months. Among 130 included patients, the median age was 58 years, 90 were male (69.2%), 30 were HBV-positive (23.1%), 102 had an Eastern Cooperative Oncology Group performance status (ECOG-PS) of 0 (78.5%), and 111 had Child–Pugh subgroup A (85.4%). Baseline CA19-9 elevation was present in 81 (62.3%) patients. Preoperative examination showed that 42 had locally advanced BTC (32.3%) and 88 had metastatic BTC (67.7%). Among the 78 patients with AR, 44 (56.4%) achieved partial response as best overall response, 5 (6.4%) achieved CR as best overall response, and 29 (37.2%) had SD lasting six months or longer. Among the 52 patients with PR, 28 (39.1%) had PD as their best overall response, while 24 (60.9%) had SD lasting less than six months. The median PFS was 6.18 months (95% CI: 5.40–6.97 months), while the median OS was 14.7 months (95% CI: 12.1–17.2 months).

### Clinical Characteristics of PR and AR

**Fig. 2:**
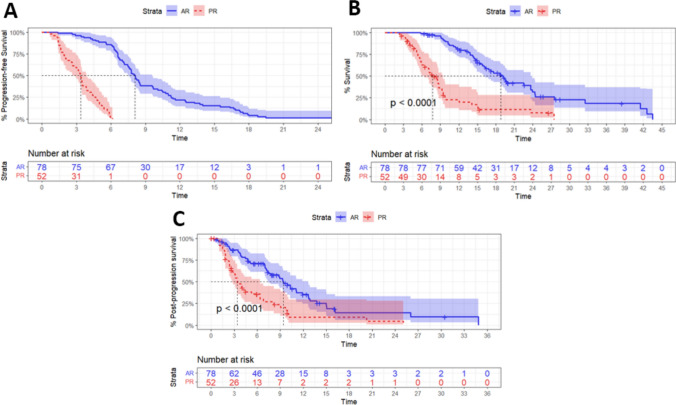
Outcomes of the cohort. **A** PFS, **B** OS, and **C** PPS of patients discriminated according to resistance type. PFS, progression-free survival; OS, overall survival; PPS, post-progression survival; CI, confidence interval; PR, primary resistance; AR, acquired resistance

The median PFS was 8.08 months in patients with AR and 3.37 months in those with PR (Fig. 2A). Most (78.2%) of the patients with AR developed resistance within one year, while all of them progressed within two years. Patients with AR had a significantly longer OS compared to those with PR (Fig. 2B). The median OS for patients with PR was 7.73 months, with a one-year survival rate of 22.7%. In contrast, patients with AR had a median OS of 18.8 months, with a one-year survival rate of 79.8%. Notably, median PPS also differed significantly between patient subgroups (9.40 months for patients with AR vs. 3.40 months for patients with PR; *P* < 0.001; Fig. 2C), which underscores a markedly better responsiveness to further interventions in AR patients compared to those with PR.

Patients with AR showed no significant differences compared to those with PR regarding age, sex, HBV infection, ECOG-PS, Child–Pugh group, primary tumor location, tumor status, or first-line ICI regimens (Table 2). Patients with AR were less common to have elevated CA19-9 level (*P* = 0.016) and were more frequently received combined target therapy (*P* = 0.006).

**Table 2:** Baseline characteristics of patients by resistance type

Characteristics	Acquired resistance (n = 80, %)	Primary resistance (n = 52, %)	*P* value
Age median (IQR), years	58 [50–64]	58 [50.75–65.75]	0.677
Sex			0.438
Male	56 (71.8)	34 (65.4)	
Female	22 (28.2)	18 (34.6)	
HBV infection			0.671
Yes	17 (21.8)	13 (25.0)	
No	61 (78.2)	39 (75.0)	
ECOG-PS			0.155
0	64 (82.1)	38 (73.1)	
1	12 (15.4)	14 (26.9)	
2	2 (2.6)	0 (0.0)	
Child–Pugh subgroup			0.478
A	68 (87.2)	43 (82.7)	
B	10 (12.8)	9 (17.3)	
Primary tumor location			0.180
ICC	55 (70.5)	44 (84.6)	
GBC	15 (19.2)	5 (9.6)	
ECC	8 (10.3)	3 (5.8)	
Tumor status			0.400
Locally advanced	23 (29.5)	19 (36.5)	
Metastatic	55 (70.5)	33 (63.5)	
Elevated CA19-9 level**			**0.016**
Yes	44 (61.1)	37 (82.2)	
No	28 (38.9)	8 (17.8)	
Not available	8	7	
ICI regimen			0.707
aPD-1	65 (83.3)	42 (80.8)	
aPD-L1	13 (16.7)	10 (19.2)	
Combination with target therapy			**0.006**
Yes	56 (71.8)	25 (48.1)	
No	22 (28.2)	27 (51.9)	

^*^ Tumor showing features of both moderately and poorly differentiated tumors

^**^ CA19-9 level exceeding 37U/ml

IQR, interquartile range; HBV, hepatitis B virus; ICC, intrahepatic cholangiocarcinoma, ECC, extrahepatic cholangiocarcinoma; GBC, gallbladder cancer; ECOG-PS, Eastern Cooperative Oncology Group Performance Status; CA19-9, carbohydrate antigen 19–9; aPD-1, anti-programmed cell death protein 1 antibody; aPD-L1, anti-programmed cell death ligand 1 antibody

Patients showed changes in baseline characteristics at the time of progression (Supplementary Table 1A). Both patients with AR and PR suffered a significant deterioration in ECOG-PS at the time of progression (PR: initial ECOG-PS 0–1–2: 73.1%–26.9%–0.0% vs. ECOG-PS upon progression: 46.2%–46.2%–7.7%, *P* = 0.007; AR: initial ECOG-PS 0–1–2: 82.1%–15.4%–2.6% vs. ECOG-PS upon progression: 55.1%–32.1%–12.8%, *P* = 0.001). Patients with AR showed no significant differences compared to those with PR regarding ECOG status, Child–Pugh group, or tumor status upon progression. Patients with AR were less common to have elevated CA19-9 level (P = 0.027) upon progression.

Among 78 patients with AR, the most common site of progression was the liver (64.1%), followed by lymph nodes (21.8%), bones (17.9%), lungs (6.4%), and bile duct system (5.1%). Among 52 patients with PR, the most common site of progression was the liver (75.0%), followed by lymph nodes (28.8%), bones (9.6%), lungs (7.7%), and vessel tumor thrombus (7.7%). No significant differences were observed between AR and PR patients according to sites of progression or progression patterns (Supplementary Table 1B). The occurrence of new lesions, multi-site progression, or systemic progression was not associated with a significant difference in post-progression survival, neither in all patients developing resistance nor in patients developing PR or AR (Supplementary Fig. 1).

### Post-resistance management and outcomes

A total of 103 patients received antitumor treatments following disease progression, including chemotherapy, LRT, targeted therapy, and immunotherapy. Among the 78 patients with AR, 63 received post-resistance antitumor treatments, while 40 of the 52 patients with PR received such treatments. The details of all subsequent therapies are summarized in Supplementary Table 2.

**Fig. 3:**
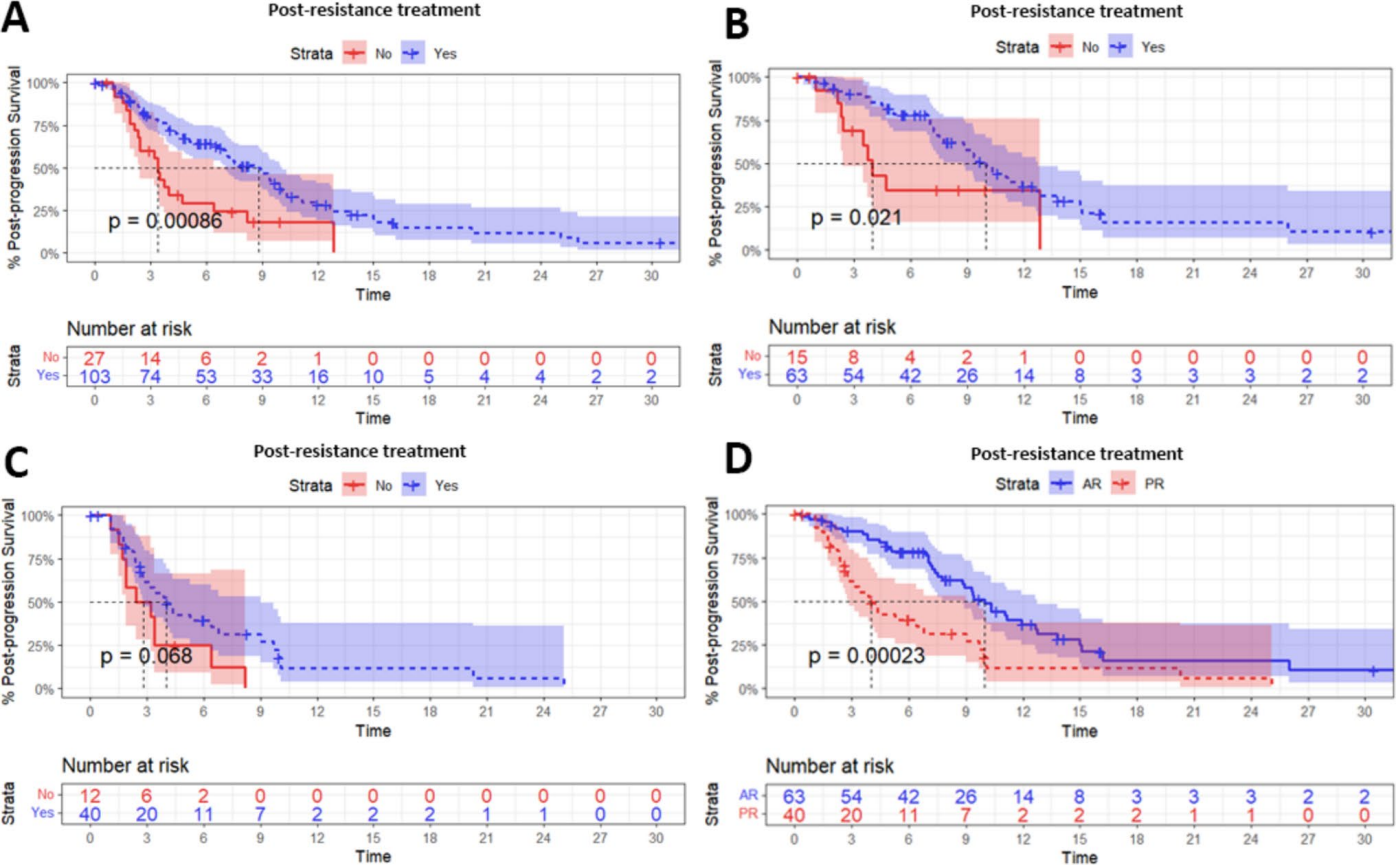
Post-resistance management outcomes. PPS of (A) all patients (B) patients with AR and (C) patients with PR that developed resistance discriminated according to post-resistance management. (D) PPS of all patients receiving active post-resistance treatment discriminated according to resistance type. PPS, post-progression survival; AR, acquired resistance; PR, primary resistance; CI, confidence interval

Patients who underwent further antitumor treatments after developing resistance demonstrated significantly improved PPS compared to those who received best supportive care (Fig. 3A; mPPS 8.80 months vs. 3.40 months; *P* < 0.001). Such benefit was observed in both AR (Fig. 3B, mPPS 9.97 months vs. 3.97 months; *P* = 0.021) and PR (Fig. 3C, mPPS 4.00 vs. 2.80 months; *P* = 0.068) subgroups. Among all patients receiving post-resistance treatment, those with AR received additional survival benefit when compared to those with PR (Table 3D; AR: mPPS 9.97 months vs. PR: mPPS 4.00 months; *P* < 0.001).

**Fig. 4:**
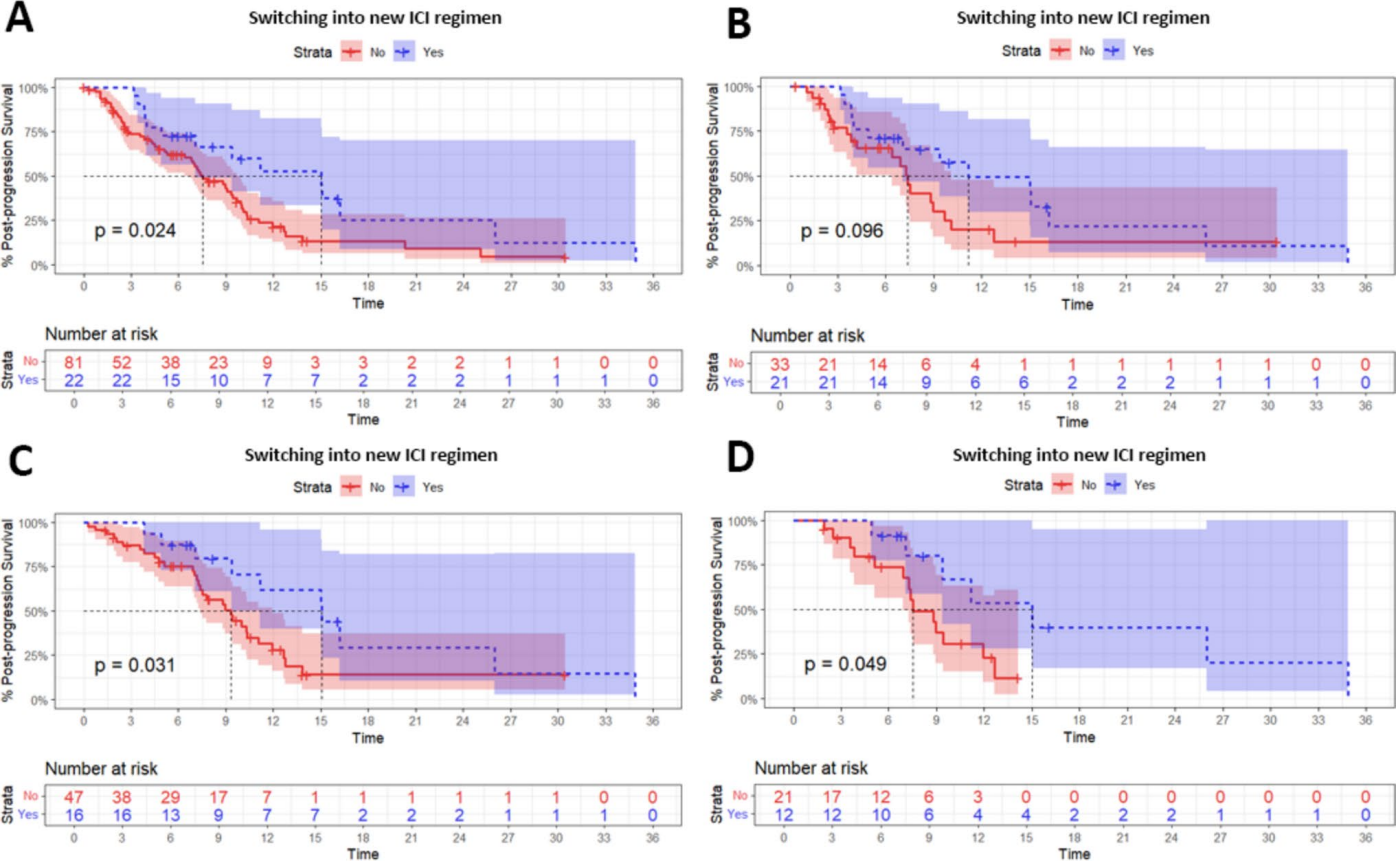
Post-progression outcomes of changing immunotherapy regimen. Post-progression survival of (A, B) all patients and (C, D) patients with AR receiving post-resistance antitumor treatment according to immunotherapy strategies before (A, C) and after (B, D) 1:2 PS matching. PPS, post-progression survival; AR, acquired resistance; PR, primary resistance; CI, confidence interval; ICI, immune checkpoint inhibitor; PS, propensity score

Post-progression survival of all patients receiving antitumor treatments after resistance was compared according to immunotherapy strategies and resistance types (Supplementary Fig. 2). Among all patients with resistance, switching into new ICI regimen after resistance significantly prolonged patients’ PPS when compared with those continuing or stopping index ICI therapy (Fig. 4A, mPPS 15.0 vs. 7.50 months, *P* = 0.024). Such strategy showed a tendency of prolonged PPS after performing a 1:2 ratio propensity score matching (Fig. 4B and Supplementary Tables 3 and 4, mPPS 11.1 vs. 7.37 months, *P* = 0.096). No significant differences were observed between patients with different immunotherapy strategies in terms of clinical characteristics and post-resistance combination treatment (Supplementary Tables 3 and 4). Other strategies, including switching into new target therapy regimen, switching into non-gemcitabine-based chemotherapy regimens, or adding LRT, demonstrated no significant impact to PPS among all patients receiving post-resistance treatment.

Specifically, among patients developing AR, switching into new ICI regimen after resistance brought significant benefit to PPS compared with other antitumor treatments (Fig. 4C, mPPS 15.1 vs. 9.30 months, *P* = 0.031). The significant difference in PPS remained even after performing a 1:2 ratio propensity score matching (Fig. 4D and Supplementary Tables 5 and 6, mPPS 15.0 vs. 9.37 months, *P* = 0.049). No significant differences were observed between AR patients with different immunotherapy strategies in terms of clinical characteristics and post-resistance combination treatment (Supplementary Tables 5 and 6). Other regimen modifications involving target therapy, chemotherapy, or LRT demonstrated no significant impact to PPS among patients with AR receiving post-resistance treatment (Supplementary Fig. 3).

Among patients developing PR, switching into new ICI regimen after resistance brought no benefit to PPS compared with other antitumor treatments (Supplementary Fig. 4A, mPPS 3.93 vs. 4.20 months, *P* = 0.63). Nor did switching into new target therapy or chemotherapy regimens (Supplementary Fig. 4B and C). Adding LRT after PR showed potential benefit to PPS compared with receiving systemic therapy only (Supplementary Fig. 4D, mPPS 9.03 months vs. 3.08 months, P = 0.057).

Univariate and multivariate Cox regression analyses on the entire cohort further confirmed the impact of resistance type to survival. (Supplementary Tables 7 and 8). Developing PR (HR = 2.24; 95% CI: 1.37–3.69; *P* = 0.001) and initial ECOG-PS ≥ 1 (HR = 1.83; 95% CI: 1.05–3.19; *P* = 0.034) emerged as strong independent risk factors. Subgroup univariate and multivariate Cox regression analyses by resistance subgroups are presented in Supplementary Tables 9–11. Among patients with AR, changing ICI regimen (HR = 0.41; 95% CI: 0.17–0.98; *P* = 0.044) emerged as an independent protective factor, while elevation of CA19-9 level upon progression (HR = 2.07; 95% CI: 1.05–4.06; *P* = 0.035) emerged as an independent risk factor. Among patients with PR, receiving post-resistance LRT (HR = 0.36; 95% CI: 0.15–0.87; *P* = 0.023) and changing target regimen after resistance (HR = 0.39; 95% CI: 0.17–0.92; *P* = 0.031) emerged as independent protective factors.

A total of 19 patients who were determined to have disease progression according to RECIST 1.1 continued their initial combination therapy. Their post-progression response assessments were reevaluated based on the iRECIST criteria, and two patients were retrospectively determined to have pseudoprogression (Supplementary Fig. 5). Both patients were initially classified as AR according to RECIST 1.1 criteria (one with a best overall response of CR and the other with SD). At the data cutoff of this study, both patients remained alive.

## Discussion

To the best of our knowledge, this study is the first to characterize the subsequent progression patterns, managements, and long-term outcomes of patients with uBTC developing resistance to first-line of chemotherapy–immunotherapy combinations in the real-world setting. We found that AR patients exhibited significantly longer mPPS compared to PR patients (9.40 vs. 3.40 months), highlighting important differences in biological behavior and therapeutic vulnerabilities after resistance. Following progression, the majority of patients received subsequent therapies, which were associated with a significant survival advantage regardless of progression type, especially among AR patients. Specifically, adjusting immunotherapy significantly improved outcomes in AR patients, suggesting the persistence of a partially active immune microenvironment. In contrast, PR patients did not benefit from adjusting immunotherapy, but showed improved survival with LRT or adjustments in targeted agents. These findings underscore the need for tailored post-progression strategies based on the underlying resistance patterns.

Despite the establishment of combinations of chemotherapy and immunotherapy regimens as the standard first-line treatment for uBTC, as supported by the TOPAZ-1 and KEYNOTE-966 trials, clinical benefits remain limited^[[3,4]]^. Our cohort showed that 67.3% of patients failed to achieve a durable response. Elevated CA19-9 levels were more frequently observed in these patients, indicating its potential role as a biomarker for early identification of poor responders. Among those who achieved a durable response, the combination of GEMOX chemotherapy and lenvatinib-based targeted therapy appeared to contribute to improved outcomes, aligning with evidence from recent phase II clinical trials^[[5]]^. Despite achieving initial disease control for at least 6–8 months, 80 patients (66.7%) with durable response eventually developed subsequent progression. Currently, no clinicopathological features have been identified to predict post-response progression. Therefore, pathological analysis of lesions at progression may provide critical insights into immune escape mechanisms and guide the development of strategies to overcome resistance.

Interestingly, in contrast with experiences in other type of tumors, we observed no significant differences in the progression patterns between AR and PR patients^[[26–28]]^. In both groups, multi-site progression was predominant, with the liver as the most common metastatic site, although extrahepatic dissemination was also frequent^[[29]]^. This reflects the aggressive biological nature of BTC, characterized by early lymphatic spread and longitudinal dissemination along the biliary tract^[[1]]^. Such biological behavior imposes substantial challenges on post-progression management and influences the selection of subsequent therapies.

Most patients received subsequent therapies after tumor progression and achieved significant survival benefits, regardless of their progression patterns. Notably, patients with AR experienced greater survival benefits compared to those with PR. Further analysis revealed that AR patients significantly benefited from changing ICI therapy regimens, whereas PR patients did not. Previous studies have shown that, unlike PR tumors, AR tumors largely retain or even enhance inflammatory features rather than displaying the immune-excluded or immune-desert phenotypes typical of PR tumors^[[30]]^. In AR tumors, significant upregulation of IFN-γ suggests the persistence, though insufficient, of antitumor immune responses^[[31,32]]^. Additionally, CD8⁺ T cell exhaustion under PD-1 blockade has been observed in AR patients in multiple cancer models^[[11,33]]^. In various malignancies, immunotherapy rechallenge after the development of resistance has demonstrated favorable safety profiles and certain degrees of efficacy^[[34–36]]^. Thus, switching immunotherapy regimens may help remodel and rescue antitumor immunity, contributing to prolonged survival in AR patients. Moreover, CAR-T therapies and strategies targeting the IFN-γ signaling pathway have shown promising efficacy in AR patients with other malignancies, indicating potential applicability in uBTC^[[32,37]]^. In contrast, PR tumors often harbor mutations in immunotherapy-resistant genes such as CDKN2A, conferring an inherently immunosuppressive microenvironment^[[38]]^. Consequently, PR patients derive little benefit from switching immunotherapy targets but may respond better to alternative targeted therapies such as CDK4/6 inhibitors like palbociclib^[[39]]^. Our findings support this approach, although the optimal post-progression targeted strategies require further investigation.

Furthermore, this study found that AR patients did not benefit from LRT, whereas survival in PR patients was potentially improved by LRT. LRT, such as radiotherapy, effectively elicit an abscopal effect provoking systemic immune response and synergizing with ICI therapies^[[40,41]]^. Studies in other type of tumors support the addition of locoregional treatments after systemic failure, which may delay resistance progression^[[42]]^. This finding provides important guidance for post-progression treatment strategies in PR patients with oligoprogression.

Although frequently observed, pseudoprogression in biliary tract cancer remains poorly characterized and lacks systematic investigation^[[43]]^. Our study confirms that a subset of patients with biliary tract cancer undergoing chemo-immunotherapy may experience pseudoprogression, and achieve durable clinical response subsequently. It remains a challenge to investigate pseudoprogression in clinical setting, as patients typically seek to switch therapies promptly upon disease progression, while those experiencing pseudoprogression may also derive benefit from more active post-progression managements. Further investigation is needed to balance rapid intervention upon real resistance with the accurate identification of pseudoprogression.

Our study has several limitations that require acknowledgment. First, it is crucial to recognize that our analyses are retrospective in nature, which introduces the possibility of information bias, including selection bias, recall bias, or misclassification bias. Second, heterogeneity in both the immunotherapy combinations among durable responders and the subsequent systemic therapies may have introduced confounding effects on the analysis of post-resistance management and outcomes. Third, the limited size of our study population prevented us from conducting adequately powered comparisons between the different subgroups. Some clinical characteristics that may have potential implications for survival, such as patients’ diet and nutritional status, were not recorded in this study. Despite these limitations, our study’s strength lies in being the first investigation, to the best of our knowledge, that comprehensively characterizes the progression patterns, dispositions, and outcomes of real-world durable responders with advanced BTC treated with immunotherapies.

## Conclusion

To our knowledge, this is the first clinical study focusing on the characteristics, prognosis, and management of resistance to uBTC first-line ICI–chemotherapy combination. Our findings identified a relatively high prevalence of resistance in real-world setting, while CA19-9 being a potential biomarker of primary resistance. Patients with both AR and PR commonly experience deterioration in general condition and systemic progression. For post-resistance managements, changing ICI therapy regimen after first-line resistance brings significant survival benefit in patients with AR but not those with PR. Changing target therapy regimens and LRT after first-line resistance brings potential benefit in patients with PR, but not those with AR. Our study cast new light on identification of resistance and precise post-resistance management in patients with uBTC receiving ICI–chemotherapy combination.

## Supplementary Information

Below is the link to the electronic supplementary material.

Supplementary file1 (DOCX 1287 KB)

## Data Availability

All data relevant to the study are included in the article or uploaded as online supplementary material. Further inquiries of the original dataset can be directed to the corresponding author.

## Abbreviations

aCTLA-4: Anti-cytotoxic T lymphocyte-associated antigen-4 antibody
aPD-1: Anti-programmed cell death protein 1 antibody
aPD-L1: Anti-programmed cell death ligand 1 antibody
AR: Acquired resistance
CA19-9: Carbohydrate antigen 19–9
CI: Confidence interval
CPD: Confirmed progression
CR: Complete response
ECC: Extrahepatic cholangiocarcinoma
ECOG: Eastern Cooperative Oncology Group
GBC: Gallbladder cancer
GC: Gemcitabine plus cisplatin
GEMOX: Gemcitabine plus oxaliplatin
HBV: Hepatitis B virus
“I”: Indicates immune responses assigned using iRECIST
ICC: Intrahepatic cholangiocarcinoma
ICI: Immune checkpoint inhibitor
IQR: Interquartile range
LRT: Local regional therapy
OS: Overall survival
PD: Progressive disease
PFS: Progression-free survival
PPS: Post-progression survival
PR: Primary resistance
RECIST: Response evaluation criteria in solid tumors
SD: Stable disease
UPD: Unconfirmed progression
uBTC: Unresectable biliary tract carcinoma
