# Supplementary material for: Immunotherapy change overcomes acquired resistance after chemo-immunotherapy in unresectable biliary tract cancer
Source: Cancer Immunol Immunother. 2025 Sep 29;74(10):320. doi: 10.1007/s00262-025-04181-2 (PMC12480270; doi:10.1007/s00262-025-04181-2)
Supplement: Supplementary file 1 — Supplementary file1 (DOCX 1287 KB) [file 262_2025_4181_MOESM1_ESM.docx]

**Supplementary materials**

Supplementary Table 1

(A) Clinical characteristics upon progression of patients with AR and PR

| **Clinical characteristics**  **upon progression** | **Acquired resistance (n=78, %)** | **Primary resistance (n=52, %)** | **P value** |
| --- | --- | --- | --- |
| **Tumour status** |  |  | 0.522 |
| Locally advanced | 58 (74.4) | 36 (69.2) |  |
| Metastatic | 20 (25.6) | 16 (30.8) |  |
| **Elevated CA19-9 level*** |  |  | **0.027** |
| Yes | 42 (60.0) | 37 (83.7) |  |
| No | 31 (40.0) | 11 (16.3) |  |
| Not available | 5 | 4 |  |
| **ECOG PS** |  |  | 0.235 |
| 0 | 43 (55.1) | 24 (46.2) |  |
| 1 | 25 (32.1) | 24 (46.2) |  |
| 2 | 10 (12.8) | 4 (7.7) |  |
| **Child-Pugh subgroup** |  |  |  |
| A | 60 (76.9) | 40 (76.9) | >0.999 |
| B | 18 (23.1) | 12 (23.1) |  |

* CA19-9 level exceeding 37U/ml.

(B) Progression patterns of patients with AR and PR

| **Patterns of progression** | **Acquired resistance (n=78, %)** | **Primary resistance (n=52, %)** | **P value** |
| --- | --- | --- | --- |
| **Occurrence of new lesion** |  |  | 0.884 |
| Yes | 46 | 30 |  |
| No | 32 | 22 |  |
| **Number of** **progressed lesions*** |  |  | 0.157 |
| Oligoprogression | 32 | 15 |  |
| Systemic progression | 46 | 37 |  |
| **Number of progressed sites**** |  |  | 0.270 |
| Single-site progression | 51 | 29 |  |
| Multi-site progression | 27 | 23 |  |
| **Site of progression** |  |  |  |
| Liver | 50 (64.1) | 39 (75) | 0.190 |
| Lymph nodes | 17 (21.8) | 15 (28.8) | 0.409 |
| Bone | 14 (17.9) | 5 (9.6) | 0.188 |
| Lung | 5 (6.4) | 4 (7.7) | 0.778 |
| Peritoneum | 3 (3.8) | 3 (5.8) | 0.683 |
| Vessel tumor thrombus | 2 (2.6) | 4 (7.7) | 0.217 |
| Bile duct system | 4 (5.1) | 1 (1.9) | 0.648 |
| No progressed lesion*** | 3 (3.8) | 1 (1.9) | 0.649 |
| Others**** | 6 | 5 |  |

* Patients with continuous rise in CA19-9 level but no radiologically confirmed progressed lesion were considered as oligoprogression

** Progression in multiple lymph node groups was considered as having multiple progressed sites

*** Patients with continuous rise in CA19-9 level but no radiologically confirmed progressed lesion

**** Including 3 adrenal progression, 2 pleural progression, 2 spleen progression, 1 stomach progression, 1 kidney progression, 1 pancreas progression and 1 intracranial progression.

ECOG PS, Eastern Cooperative Oncology Group Performance Status; CA19-9, Carbohydrate antigen 19-9.

Supplementary Table 2

Post-progression management of patients according to therapy regimens

| **Treatments** | **Patients (%)** | **Acquired resistance**  **(%)** | **Primary resistance**  **(%)** |
| --- | --- | --- | --- |
| All | 130(100) | 78 (100) | 52 (100) |
| **Systemic therapies** | 93 (71.5) | 57 (73.0) | 36 (69.2) |
| 1 | 2 (1.5) | 2 (2.6) | 0 (0) |
| 2 | 7 (5.4) | 2 (2.6) | 5 (9.6) |
| 3 | 3 (2.3) | 2 (2.6) | 1 (1.9) |
| 1+2 | 9 (6.9) | 4 (5.1) | 5 (1.1) |
| 1+3 | 13 (10.0) | 8 (10.2) | 5 (9.6) |
| 2+3 | 21 (16.1) | 15 (19.2) | 6 (11.5) |
| 1+2+3 | 38 (29.2) | 24 (30.8) | 14 (26.9) |
| No systemic therapy | 37 (28.5) | 21 (26.9) | 16 (30.8) |
| **LRTs** | 39 (30.0) | 24 (30.8) | 15 (28.8) |
| TACE/HAIC | 16 (12.3) | 8 (10.2) | 8 (15.4) |
| Ablation | 6 (4.6) | 4 (5.1) | 2 (3.8) |
| Radiotherapy | 17 (13.1) | 12 (15.4) | 5 (9.6) |
| No LRT | 91 (70.0) | 54 (69.2) | 37 (71.1) |

1. Chemotherapy, 2. Target therapy, 3. Immune checkpoint inhibitor.

* 60 patients received aPD-1 ICI therapy pembrolizumab, camrelizumab, sintilimab, tislelizumab (200 mg, q3w), and toripalimab (240 mg, q3w), 14 received aPD-L1 ICI therapy devalumab (1500 mg, q3w), 1 received the PD-1/CTLA-4 inhibitor cadonilimab (6 mg/kg, q3w).

** 19 patients switched into other small-molecule tyrosine kinase inhibitors including anlotinib (10 mg qd, d1-14, every 3 weeks), regorafenib (160 mg, qd, d1-21, every 4 weeks) and apatinib (250mg, qd), 7 patients received anti-VEGF monoclonal antibody bevacizumab (5 mg/kg, q3w), 3 patients received anti-HER2 monoclonal antibody disitamab vedotin (2.0 mg/kg with a maximum dose of 120 mg, q3w), and 46 patients received lenvatinib(8 mg/day for body weight<60 kg or 12 mg/day for body weight≥ 60 kg).

*** 40 patients received gemcitabine-based chemotherapies, 22 patients received other non- gemcitabine-based chemotherapy regimens.

ICI, Immune checkpoint inhibitor; TACE/HAIC, transcatheter arterial chemoembolization/hepatic arterial infusion chemotherapy; aPD-1, anti-programmed cell death protein 1; aPD-L1, anti- Programmed cell death ligand 1; CTLA-4, cytotoxic T lymphocyte-associated antigen-4 VEGF, vascular endothelial growth factor; HER2, human epidermal growth factor receptor 2.

Supplementary Table 3

| **Clinical characteristics** | **Switching into new**  **ICI regimen**  **(n=22)** | **Continuing or stopping**  **index therapy**  **(n=81)** | **P value** |
| --- | --- | --- | --- |
| **Age ≥ 65 yrs** |  |  | 0.759 |
| Yes | 5 | 21 |  |
| No | 17 | 60 |  |
| **Sex** |  |  | 0.134 |
| Male | 13 | 61 |  |
| Female | 9 | 20 |  |
| **Primary tumour**  **location** |  |  | 0.205 |
| ICC | 18 | 63 |  |
| GBC | 4 | 9 |  |
| ECC | 0 | 9 |  |
| **Type of resistance** |  |  | 0.210 |
| Primary resistance | 6 | 34 |  |
| Acquired resistance | 16 | 47 |  |
| **ECOG PS** **upon**  **progression** |  |  | 0.528 |
| 0 | 14 | 41 |  |
| 1 | 7 | 33 |  |
| 2 | 1 | 7 |  |
| **Child-Pugh subgroup**  **upon progression** |  |  | 0.113 |
| A | 20 | 61 |  |
| B | 2 | 20 |  |
| **Tumour status upon**  **progression** |  |  | 0.456 |
| Locally advanced | 17 | 56 |  |
| Metastatic | 5 | 25 |  |
| **Elevated CA19-9 level**  **upon progression *** |  |  | 0.509 |
| Yes | 13 | 54 |  |
| No | 9 | 27 |  |
| **Occurrence of new**  **lesion** |  |  | 0.377 |
| Yes | 14 | 43 |  |
| No | 8 | 38 |  |
| **Number of progressed**  **lesions**** |  |  | 0.740 |
| Oligoprogression | 9 | 30 |  |
| Systemic progression | 13 | 51 |  |
| **Number of progressed**  **sites***** |  |  | 0.740 |
| Single-site progression | 13 | 51 |  |
| Multi-site progression | 9 | 30 |  |

(A) The clinical characteristics of all patients before propensity score matching according to immunotherapy strategies

* CA19-9 level exceeding 37U/ml.

** Patients with continuous rise in CA19-9 or CEA level but no radiologically confirmed progressed lesion were considered as oligoprogression

*** Progression in multiple lymph node groups was considered as having multiple progressed sites

(B) The post-progression combination treatment strategies of all patients before propensity score matching according to immunotherapy strategies.

| **Combination treatment strategies** | **Switching into new**  **ICI regimen**  **(n=22)** | **Continuing or stopping**  **index therapy**  **(n=81)** | **P value** |
| --- | --- | --- | --- |
| **Switching into new**  **target therapy regimen** |  |  | 0.376 |
| Yes | 11 | 32 |  |
| No | 11 | 49 |  |
| **Switching into new**  **chemotherapy regimen** |  |  | 0.805 |
| Yes | 6 | 16 |  |
| No | 16 | 61 |  |
| **Adding LRT** |  |  | 0.248 |
| Yes | 6 | 33 |  |
| No | 16 | 48 |  |

ECOG PS, Eastern Cooperative Oncology Group Performance Status; ICC, Intrahepatic Cholangiocarcinoma, ECC, Extrahepatic Cholangiocarcinoma; GBC, Gall Bladder Cancer; CA19-9, Carbohydrate antigen 19-9; LRT, locoregional therapy.

Supplementary Table 4

(A) The clinical characteristics of all patients after propensity score matching according to immunotherapy strategies

| **Clinical characteristics** | **Switching into new**  **ICI regimen**  **(n=21)** | **Continuing or stopping**  **index therapy**  **(n=33)** | **P value** |
| --- | --- | --- | --- |
| **Age ≥ 65 yrs** |  |  | 0.971 |
| Yes | 5 | 8 |  |
| No | 16 | 25 |  |
| **Sex** |  |  | 0.404 |
| Male | 13 | 24 |  |
| Female | 8 | 9 |  |
| **Primary tumour**  **location** |  |  | 0.817 |
| ICC | 18 | 29 |  |
| GBC | 3 | 4 |  |
| ECC | 0 | 0 |  |
| **Type of resistance** |  |  | 0.417 |
| Primary resistance | 6 | 13 |  |
| Acquired resistance | 15 | 20 |  |
| **ECOG PS upon**  **progression** |  |  | 0.979 |
| 0 | 13 | 20 |  |
| 1 | 7 | 11 |  |
| 2 | 1 | 2 |  |
| **Child-Pugh subgroup**  **upon progression** |  |  | 0.310 |
| A | 19 | 32 |  |
| B | 2 | 1 |  |
| **Tumour status upon**  **progression** |  |  | 0.617 |
| Locally advanced | 16 | 27 |  |
| Metastatic | 5 | 6 |  |
| **Elevated CA19-9 level**  **upon progression *** |  |  | 0.535 |
| Yes | 12 | 16 |  |
| No | 9 | 17 |  |
| **Occurrence of new**  **lesion** |  |  | 0.454 |
| Yes | 13 | 17 |  |
| No | 8 | 16 |  |
| **Number of progressed**  **lesions**** |  |  | 0.801 |
| Oligoprogression | 9 | 13 |  |
| Systemic progression | 12 | 20 |  |
| **Number of progressed**  **sites***** |  |  | 0.924 |
| Single-site progression | 13 | 20 |  |
| Multi-site progression | 8 | 13 |  |

* CA19-9 level exceeding 37U/ml.

** Patients with continuous rise in CA19-9 or CEA level but no radiologically confirmed progressed lesion were considered as oligoprogression

*** Progression in multiple lymph node groups was considered as having multiple progressed sites

(B) The post-progression combination treatment strategies of all patients after propensity score matching according to immunotherapy strategies.

| **Combination treatment strategies** | **Switching into new**  **ICI regimen**  **(n=21)** | **Continuing or stopping**  **index therapy**  **(n=33)** | **P value** |
| --- | --- | --- | --- |
| **Switching into new**  **target therapy regimen** |  |  | 0.815 |
| Yes | 7 | 10 |  |
| No | 14 | 23 |  |
| **Switching into new**  **chemotherapy regimen** |  |  | 0.892 |
| Yes | 6 | 10 |  |
| No | 15 | 23 |  |
| **Adding LRT** |  |  | 0.215 |
| Yes | 6 | 15 |  |
| No | 15 | 18 |  |

ECOG PS, Eastern Cooperative Oncology Group Performance Status; ICC, Intrahepatic Cholangiocarcinoma, ECC, Extrahepatic Cholangiocarcinoma; GBC, Gall Bladder Cancer; CA19-9, Carbohydrate antigen 19-9; LRT, locoregional therapy.

Supplementary Table 5

| **Clinical characteristics** | **Switching into new**  **ICI regimen**  **(n=16)** | **Continuing or stopping**  **index therapy**  **(n=47)** | **P value** |
| --- | --- | --- | --- |
| **Age ≥ 65 yrs** |  |  | 0.757 |
| Yes | 4 | 10 |  |
| No | 12 | 37 |  |
| **Sex** |  |  | 0.137 |
| Male | 10 | 38 |  |
| Female | 6 | 9 |  |
| **Primary tumour**  **location** |  |  | 0.249 |
| ICC | 13 | 31 |  |
| GBC | 3 | 9 |  |
| ECC | 0 | 7 |  |
| **ECOG PS upon**  **progression** |  |  | 0.192 |
| 0 | 12 | 23 |  |
| 1 | 3 | 17 |  |
| 2 | 1 | 7 |  |
| **Child-Pugh subgroup**  **upon progression** |  |  | 0.219 |
| A | 14 | 34 |  |
| B | 2 | 13 |  |
| **Tumour status upon**  **progression** |  |  | 0.479 |
| Locally advanced | 3 | 13 |  |
| Metastatic | 13 | 34 |  |
| **Elevated CA19-9 level**  **upon progression *** |  |  | 0.605 |
| Yes | 8 | 27 |  |
| No | 8 | 20 |  |
| **Occurrence of new**  **lesion** |  |  | 0.425 |
| Yes | 11 | 27 |  |
| No | 5 | 20 |  |
| **Number of progressed**  **lesions**** |  |  | 0.329 |
| Oligoprogression | 8 | 17 |  |
| Systemic progression | 8 | 30 |  |
| **Number of progressed**  **sites***** |  |  | 0.802 |
| Single-site progression | 10 | 31 |  |
| Multi-site progression | 6 | 16 |  |

(A) The clinical characteristics of AR patients before propensity score matching according to immunotherapy strategies

* CA19-9 level exceeding 37U/ml.

** Patients with continuous rise in CA19-9 or CEA level but no radiologically confirmed progressed lesion were considered as oligoprogression

*** Progression in multiple lymph node groups was considered as having multiple progressed sites

(B) The post-progression combination treatment strategies of AR patients before propensity score matching according to immunotherapy strategies.

| **Combination treatment strategies** | **Switching into new**  **ICI regimen**  **(n=16)** | **Continuing or stopping**  **index therapy**  **(n=47)** | **P value** |
| --- | --- | --- | --- |
| **Switching into new**  **target therapy regimen** |  |  | 0.459 |
| Yes | 6 | 13 |  |
| No | 10 | 34 |  |
| **Switching into new**  **chemotherapy regimen** |  |  | 0.582 |
| Yes | 3 | 12 |  |
| No | 13 | 35 |  |
| **Adding LRT** |  |  | 0.514 |
| Yes | 5 | 19 |  |
| No | 11 | 28 |  |

AR, acquired resistance; ECOG PS, Eastern Cooperative Oncology Group Performance Status; ICC, Intrahepatic Cholangiocarcinoma, ECC, Extrahepatic Cholangiocarcinoma; GBC, Gall Bladder Cancer; CA19-9, Carbohydrate antigen 19-9; LRT, locoregional therapy.

Supplementary Table 6

(A) The clinical characteristics of AR patients after propensity score matching according to immunotherapy strategies

| **Clinical characteristics** | **Switching into new**  **ICI regimen**  **(n=12)** | **Continuing or stopping**  **index therapy**  **(n=21)** | **P value** |
| --- | --- | --- | --- |
| **Age ≥ 65 yrs** |  |  | 0.865 |
| Yes | 2 | 4 |  |
| No | 10 | 17 |  |
| **Sex** |  |  | 0.775 |
| Male | 8 | 15 |  |
| Female | 4 | 6 |  |
| **Primary tumour**  **location** |  |  | 0.865 |
| ICC | 10 | 17 |  |
| GBC | 2 | 4 |  |
| ECC | 0 | 0 |  |
| **ECOG PS upon**  **progression** |  |  | 0.906 |
| 0 | 8 | 14 |  |
| 1 | 3 | 6 |  |
| 2 | 1 | 1 |  |
| **Child-Pugh subgroup**  **upon progression** |  |  | 0.679 |
| A | 11 | 20 |  |
| B | 1 | 1 |  |
| **Tumour status upon**  **progression** |  |  | 0.629 |
| Locally advanced | 2 | 5 |  |
| Metastatic | 10 | 16 |  |
| **Elevated CA19-9 level**  **upon progression *** |  |  | 0.392 |
| Yes | 5 | 12 |  |
| No | 7 | 9 |  |
| **Occurrence of new**  **lesion** |  |  | 0.840 |
| Yes | 7 | 13 |  |
| No | 5 | 8 |  |
| **Number of progressed**  **lesions**** |  |  | 0.947 |
| Oligoprogression | 5 | 9 |  |
| Systemic progression | 7 | 12 |  |
| **Number of progressed**  **sites***** |  |  | 0.687 |
| Single-site progression | 9 | 17 |  |
| Multi-site progression | 3 | 4 |  |

* CA19-9 level exceeding 37U/ml.

** Patients with continuous rise in CA19-9 or CEA level but no radiologically confirmed progressed lesion were considered as oligoprogression

*** Progression in multiple lymph node groups was considered as having multiple progressed sites

(B) The post-progression combination treatment strategies of AR patients after propensity score matching according to immunotherapy strategies.

| **Combination treatment strategies** | **Switching into new**  **ICI regimen**  **(n=12)** | **Continuing or stopping**  **index therapy**  **(n=21)** | **P value** |
| --- | --- | --- | --- |
| **Switching into new**  **target therapy regimen** |  |  | 0.690 |
| Yes | 4 | 5 |  |
| No | 8 | 16 |  |
| **Switching into new**  **chemotherapy regimen** |  |  | >0.999 |
| Yes | 2 | 5 |  |
| No | 10 | 16 |  |
| **Adding LRT** |  |  | 0.554 |
| Yes | 5 | 11 |  |
| No | 7 | 10 |  |

AR, acquired resistance; ECOG PS, Eastern Cooperative Oncology Group Performance Status; ICC, Intrahepatic Cholangiocarcinoma, ECC, Extrahepatic Cholangiocarcinoma; GBC, Gall Bladder Cancer; CA19-9, Carbohydrate antigen 19-9; LRT, locoregional therapy.

Supplementary Table 7 Single-variant Cox regression analysis on all patients developing resistance

| **Characteristics** | **HR** | **p** | **95% CI** |
| --- | --- | --- | --- |
| **Primary resistance** | **2.53** | **<0.001** | **1.64 - 3.91** |
| Age ≥ 65 yrs | 1.13 | 0.613 | 0.71 - 1.78 |
| Sex Male | 0.84 | 0.441 | 0.54 - 1.31 |
| HBV positive | 1.01 | 0.962 | 0.59 - 1.73 |
| ICC | 1.44 | 0.142 | 0.88 - 2.35 |
| ICI regimen |  |  |  |
| aPD-1 | 1 | - | - |
| aPD-L1 | 1.05 | 0.852 | 0.60 - 1.84 |
| **Combining target therapy** | **0.65** | **0.054** | **0.42 – 1.01** |
| **Child-Pugh group B** | **2.13** | **0.013** | **1.17 – 3.87** |
| **ECOG ≥1** | **1.80** | **0.015** | **1.12 – 2.88** |
| Metastatic tumor | 1.12 | 0.623 | 0.71 – 1.79 |
| Elevated CA19-9* | 1.27 | 0.330 | 0.78 – 2.08 |
| Child-Pugh group B upon  Progression | 1.41 | 0.153 | 0.88 – 2.26 |
| ECOG ≥1 upon progression | 1.25 | 0.304 | 0.82 – 1.92 |
| Metastatic tumor upon progression | 0.94 | 0.805 | 0.57 - 1.55 |
| **Elevated CA19-9* upon progression** | **1.75** | **0.024** | **1.08 - 2.86** |
| Occurrence of new lesion | 1.11 | 0.648 | 0.72 - 1.70 |
| Systemic progression | 0.86 | 0.511 | 0.56 - 1.33 |
| Multi-site progression | 0.95 | 0.808 | 0.61 - 1.46 |
| **Post-resistance treatment** | **0.43** | **0.001** | **0.25 - 0.71** |
| Changing target therapy regimen | 0.70 | 0.116 | 0.44 - 1.09 |
| Changing chemotherapy regimen | 0.96 | 0.873 | 0.55 - 1.67 |
| **Changing ICI therapy regimen** | **0.43** | **0.007** | **0.24 - 0.80** |
| Post-resistance LRT | 0.69 | 0.122 | 0.43 - 1.10 |

* CA19-9 level exceeding 37U/ml.

HBV, Hepatitis B virus; ICC, Intrahepatic Cholangiocarcinoma; ECOG, Eastern Cooperative Oncology Group; CA19-9, Carbohydrate antigen 19-9; aPD-1, Anti-programmed cell death protein 1 antibody; aPD-L1, Anti-programmed cell death ligand 1 antibody; ICI, Immune checkpoint inhibitor; LRT, local regional therapy; HR, Hazard Ratio; CI, Confidence Interval.

Supplementary Table 8 Multi-variant Cox regression analysis on all patients developing resistance.

| **Characteristics** | **HR** | **p** | **95% CI** |
| --- | --- | --- | --- |
| **Primary resistance** | **2.24** | **0.001** | **1.37 - 3.69** |
| Child-Pugh group B | 1.52 | 0.254 | 0.74 – 3.15 |
| **ECOG ≥1** | **1.83** | **0.034** | **1.05 – 3.19** |
| Combining target therapy | 0.88 | 0.638 | 0.53 - 1.48 |
| Post-resistance treatment | 0.60 | 0.133 | 0.30 – 1.17 |
| Changing ICI regimen | 0.59 | 0.106 | 0.31 - 1.12 |

ECOG, Eastern Cooperative Oncology Group; ICI, Immune checkpoint inhibitor; HR, Hazard Ratio; CI, Confidence Interval.

Supplementary Table 9 Single-variant Cox regression analysis on patients with acquired resistance.

| **Characteristics** | **HR** | **p** | **95% CI** |
| --- | --- | --- | --- |
| Age ≥ 65 yrs | 0.93 | 0.836 | 0.49 - 1.77 |
| Sex Male | 1.08 | 0.807 | 0.58 – 2.02 |
| HBV positive | 0.88 | 0.739 | 0.43 – 1.83 |
| ICC | 1.20 | 0.562 | 0.65 – 2.22 |
| ICI regimen |  |  |  |
| aPD-1 | 1 | - | - |
| aPD-L1 | 1.00 | 0.994 | 0.47 – 2.15 |
| Combining target therapy | 0.83 | 0.557 | 0.44 – 1.55 |
| **Child-Pugh group B** | **2.94** | **0.014** | **1.25 – 6.91** |
| ECOG ≥1 | 1.57 | 0.179 | 0.81 – 3.02 |
| Metastatic tumor | 1.29 | 0.447 | 0.67 – 2.49 |
| Elevated CA19-9* | 1.31 | 0.388 | 0.71 – 2.43 |
| **Child-Pugh group B upon**  **Progression** | **1.79** | **0.073** | **0.95 – 3.39** |
| ECOG ≥1 upon progression | 1.33 | 0.335 | 0.75 – 2.36 |
| Metastatic tumor upon progression | 1.08 | 0.832 | 0.53 – 2.19 |
| **Elevated CA19-9* upon progression** | **2.11** | **0.023** | **1.11 – 4.02** |
| Occurrence of new lesion | 1.18 | 0.583 | 0.66 – 2.11 |
| Systemic progression | 0.66 | 0.158 | 0.37 – 1.17 |
| Multi-site progression | 0.71 | 0.280 | 0.38 – 1.32 |
| **Post-resistance treatment** | **0.43** | **0.026** | **0.20 – 0.90** |
| Changing target therapy regimen | 0.79 | 0.454 | 0.43 – 1.45 |
| Changing chemotherapy regimen | 0.93 | 0.863 | 0.43 – 2.01 |
| **Changing ICI therapy regimen** | **0.38** | **0.015** | **0.17 – 0.83** |
| Post-resistance LRT | 0.92 | 0.781 | 0.50 – 1.69 |

* CA19-9 level exceeding 37U/ml.

HBV, Hepatitis B virus; ICC, Intrahepatic Cholangiocarcinoma; ECOG, Eastern Cooperative Oncology Group; CA19-9, Carbohydrate antigen 19-9; aPD-1, Anti-programmed cell death protein 1 antibody; aPD-L1, Anti-programmed cell death ligand 1 antibody; ICI, Immune checkpoint inhibitor; LRT, local regional therapy; HR, Hazard Ratio; CI, Confidence Interval.

Supplementary Table 10 Single-variant Cox regression analysis on patients with primary resistance.

| **Characteristics** | **HR** | **p** | **95% CI** |
| --- | --- | --- | --- |
| Age ≥ 65 yrs | 1.56 | 0.196 | 0.79 – 3.06 |
| Sex Male | 0.65 | 0.194 | 0.34 - 1.24 |
| HBV positive | 1.13 | 0.753 | 0.52 – 2.49 |
| ICC | 1.37 | 0.459 | 0.60 – 3.12 |
| ICI regimen |  |  |  |
| aPD-1 | 1 | - | - |
| aPD-L1 | 1.10 | 0.825 | 0.48 – 2.54 |
| Combining target therapy | 0.66 | 0.199 | 0.42 – 1.01 |
| Child-Pugh group B | 2.13 | 0.388 | 0.34 – 1.25 |
| **ECOG ≥1** | **2.32** | **0.024** | **1.12 – 4.80** |
| Metastatic tumor | 1.11 | 0.769 | 0.56 – 2.20 |
| Elevated CA19-9* | 0.59 | 0.225 | 0.25 – 1.39 |
| Child-Pugh group B upon  Progression | 1.44 | 0.388 | 0.63 – 3.33 |
| ECOG ≥1 upon progression | 1.08 | 0.824 | 0.56 – 2.06 |
| Metastatic tumor upon progression | 0.78 | 0.503 | 0.37 - 1.73 |
| Elevated CA19-9* upon progression | 0.80 | 0.577 | 1.08 - 2.86 |
| Occurrence of new lesion | 0.97 | 0.927 | 0.51 - 1.86 |
| Systemic progression | 1.12 | 0.760 | 0.54 – 2.31 |
| Multi-site progression | 1.01 | 0.971 | 0.53 - 1.94 |
| **Post-resistance treatment** | **0.51** | **0.069** | **0.25 – 1.05** |
| **Changing target therapy regimen** | **0.43** | **0.022** | **0.21 – 0.88** |
| Changing chemotherapy regimen | 0.99 | 0.974 | 0.43 – 2.25 |
| Changing ICI therapy regimen | 0.65 | 0.426 | 0.23 – 1.86 |
| **Post-resistance LRT** | **0.40** | **0.022** | **0.18 – 0.88** |

* CA19-9 level exceeding 37U/ml.

HBV, Hepatitis B virus; ICC, Intrahepatic Cholangiocarcinoma; ECOG, Eastern Cooperative Oncology Group; CA19-9, Carbohydrate antigen 19-9; aPD-1, Anti-programmed cell death protein 1 antibody; aPD-L1, Anti-programmed cell death ligand 1 antibody; ICI, Immune checkpoint inhibitor; LRT, local regional therapy; HR, Hazard Ratio; CI, Confidence Interval.

Supplementary Table 11

(A). Multi-variant Cox regression analysis on patients with acquired resistance.

| **Characteristics** | **HR** | **p** | **95% CI** |
| --- | --- | --- | --- |
| Child-Pugh group B | 2.00 | 0.183 | 0.72 – 5.59 |
| Child-Pugh group B  upon progression | 1.24 | 0.563 | 0.59 – 2.60 |
| **Elevated CA19-9* upon progression** | **2.07** | **0.035** | **1.05 – 4.06** |
| Post-resistance treatment | 0.93 | 0.901 | 0.31 – 2.80 |
| **Changing ICI regimen** | **0.41** | **0.044** | **0.17 - 0.98** |

(B). Multi-variant Cox regression analysis on patients with primary resistance.

| **Characteristics** | **HR** | **p** | **95% CI** |
| --- | --- | --- | --- |
| ECOG ≥1 | 1.97 | 0.075 | 0.93 – 4.14 |
| Post-resistance treatment | 1.12 | 0.792 | 0.48 – 2.59 |
| **Changing target regimen** | **0.39** | **0.031** | **0.17 – 0.92** |
| **Post-resistance LRT** | **0.36** | **0.023** | **0.15 – 0.87** |

* CA19-9 level exceeding 37U/ml.

HBV, Hepatitis B virus; ICC, Intrahepatic Cholangiocarcinoma; ECOG, Eastern Cooperative Oncology Group; CA19-9, Carbohydrate antigen 19-9; aPD-1, Anti-programmed cell death protein 1 antibody; aPD-L1, Anti-programmed cell death ligand 1 antibody; ICI, Immune checkpoint inhibitor; LRT, local regional therapy; HR, Hazard Ratio; CI, Confidence Interval.


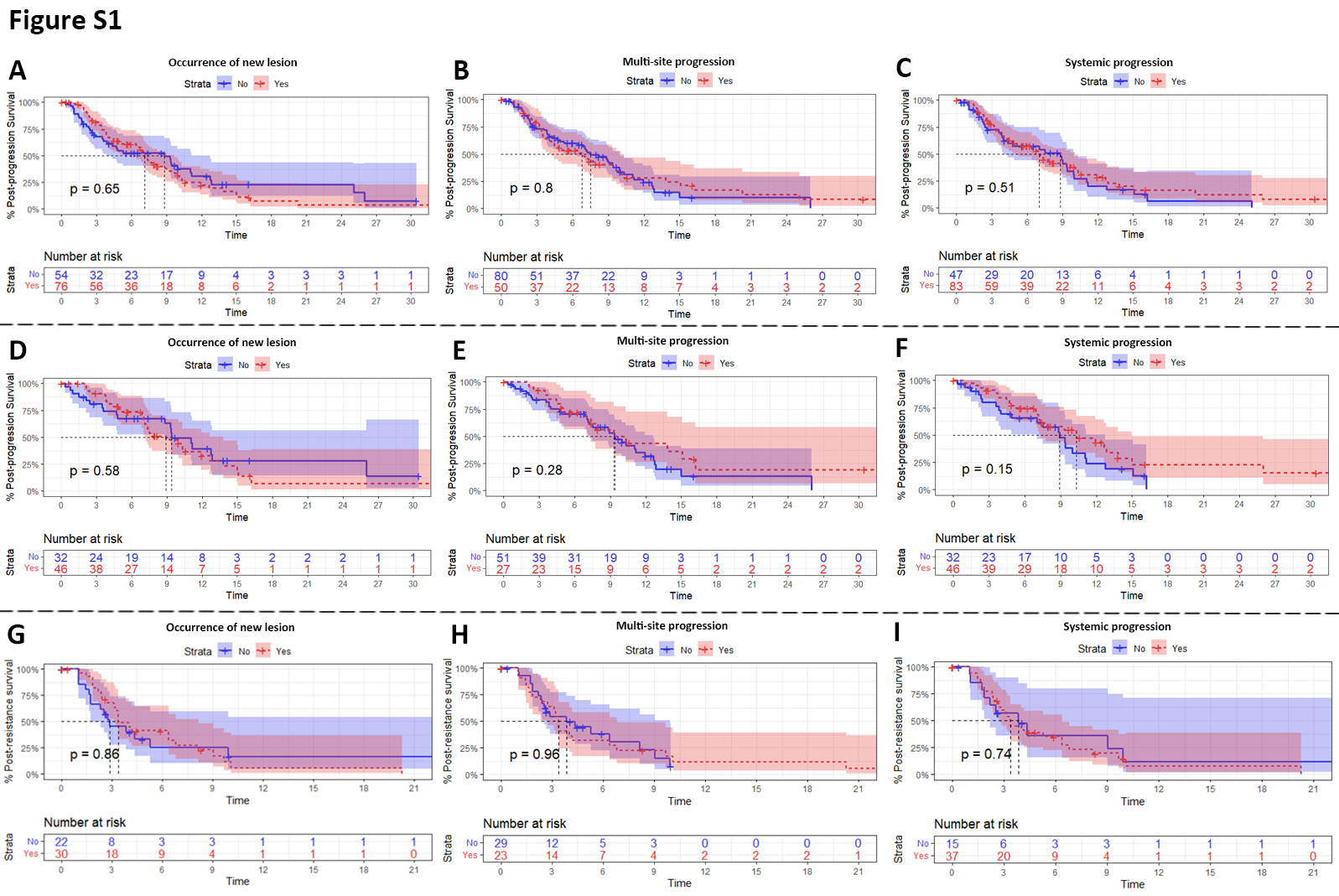
Supplementary Fig. 1. Post-progression outcomes by progression patterns.

(A, B, C) Post-progression survival of all patients developing resistance according to (A) occurrence of new lesions, (B) having multi-site progression and (C) having systemic progression. No significant difference was observed (occurrence of new lesions: Yes: mPPS 7.13 months vs. No: mPPS 8.80 months; multi-site progression: Yes: mPPS 6.80 months vs. No: mPPS 7.50 months; systemic progression: Yes: mPPS 7.03 months vs. No: mPPS 8.80 months).

(D, E, F) Post-progression survival of patients with AR according to (D) occurrence of new lesions, (E) having multi-site progression and (F) having systemic progression. No significant difference was observed (occurrence of new lesions: Yes: mPPS 8.93 months vs. No: mPPS 9.40 months; multi-site progression: Yes: mPPS 9.30 months vs. No: mPPS 9.40 months; systemic progression: Yes: mPPS 10.33 months vs. No: mPPS 8.93 months).

(G, H, I) Post-progression survival of patients with PR according to (G) occurrence of new lesions, (H) having multi-site progression and (I) having systemic progression. No significant difference was observed (occurrence of new lesions: Yes: mPPS 3.40 months vs. No: mPPS 2.87 months; multi-site progression: Yes: mPPS 3.40 months vs. No: mPPS 3.87 months; systemic progression: Yes: mPPS 3.40 months vs. No: mPPS 3.87 months).

AR, acquired resistance; PR; primary resistance; mPPS: median post-progression survival.


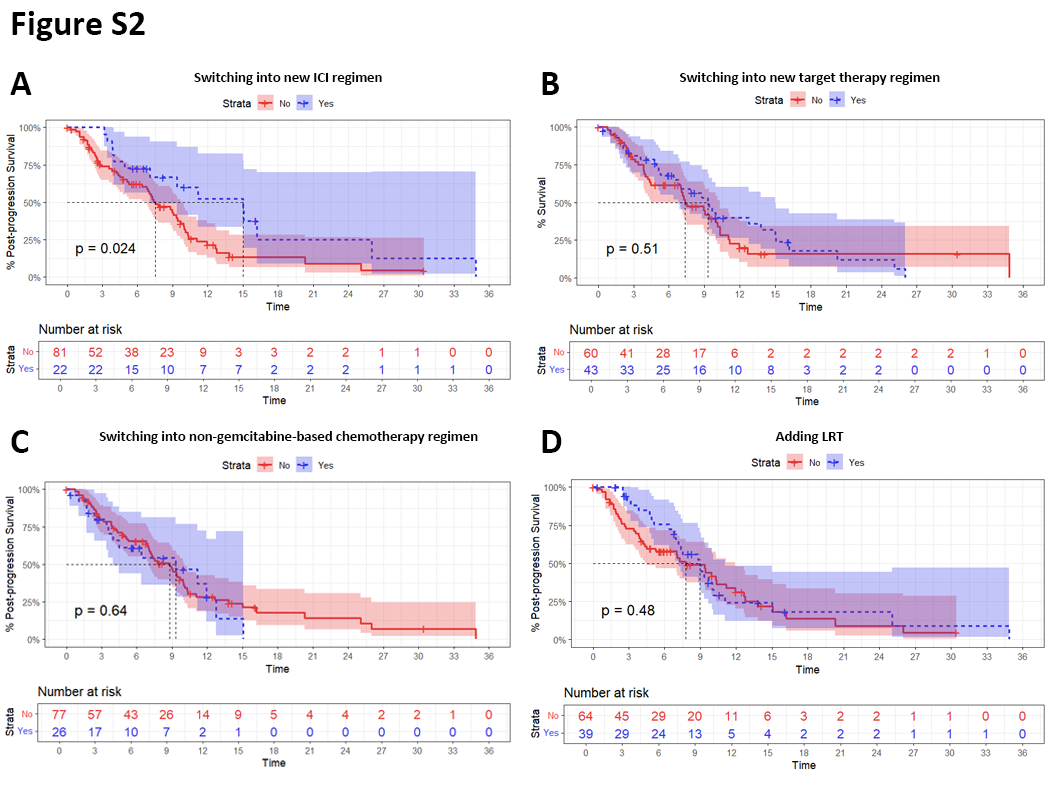
Supplementary Fig. 2.

Post-progression survival of all patients receiving anti-tumour treatment after resistance according to (A) immunotherapy strategies, (B) target therapy strategies, (C) chemotherapy strategies, and (D) LRT strategies.

Significant difference in mPPS was observed between patients who switched into new ICI regimen and those who continued or stopped index therapy (mPPS 15.0 months vs. 7.50 months, p=0.024). No significant difference was observed between patients who switched into new target therapy regimen and those who continued or stopped index therapy (mPPS 9.30 months vs. 7.37 months, p=0.51), those who switched into non-gemcitabine-based regimen and those who continued or stopped gemcitabine-based chemotherapy (mPPS 9.30 months vs. 8.80 months, p=0.64), or those who added LRT after resistance and those who received systemic therapy only (mPPS 8.93 months vs. 7.77 months, p=0.48)

mPPS: median post-progression survival; ICI, immune checkpoint inhibitor; LRT, local regional therapy.


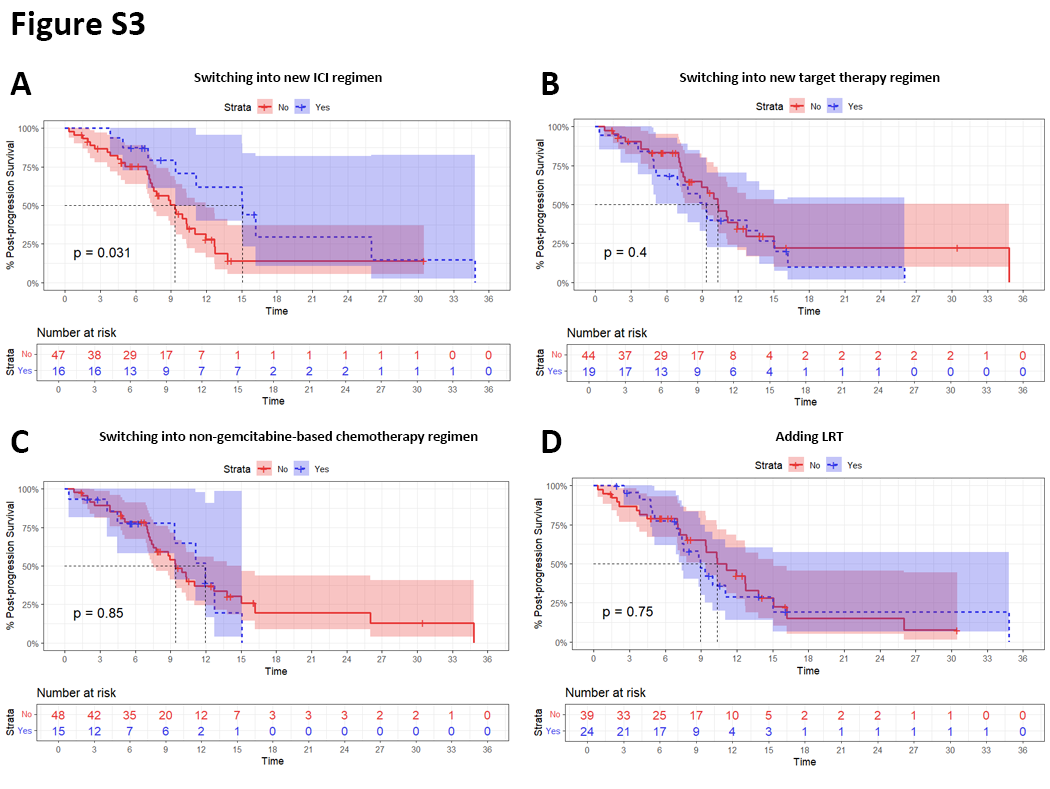


Supplementary Fig. 3.

Post-progression survival of patients receiving anti-tumour treatment after AR according to (A) immunotherapy strategies, (B) target therapy strategies, (C) chemotherapy strategies, and (D) LRT strategies.

Significant difference in mPPS was observed between AR patients who switched into new ICI regimen and those who continued or stopped index therapy (mPPS 15.1 months vs. 9.30 months, p=0.031). No significant difference was observed between patients who switched into new target therapy regimen and those who continued or stopped index therapy (mPPS 9.30 months vs. 10.30 months, p=0.40), those who switched into non-gemcitabine-based regimen and those who continued or stopped gemcitabine-based chemotherapy (mPPS 11.90 months vs. 9.40 months, p=0.85), or those who added LRT after resistance and those who received systemic therapy only (mPPS 8.93 months vs. 10.33 months, p=0.75)

mPPS: median post-progression survival; ICI, immune checkpoint inhibitor; LRT, local regional therapy.


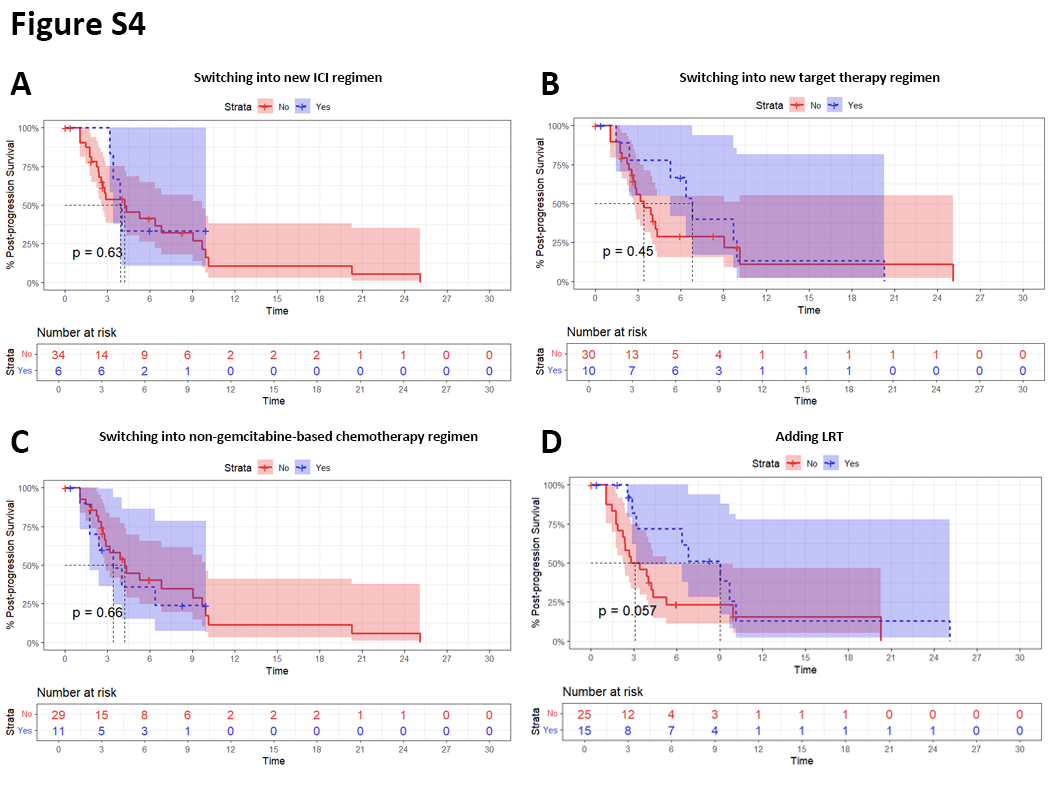
Supplementary Fig. 4.

Post-progression survival of patients receiving anti-tumour treatment after PR according to (A) immunotherapy strategies, (B) target therapy strategies, (C) chemotherapy strategies, and (D) LRT strategies.

No significant difference was observed between PR patients who switched into new ICI regimen and those who continued or stopped index therapy (mPPS 3.93 months vs. 4.20 months, p=0.63), those who switched into new target therapy regimen and those who continued or stopped index therapy (mPPS 6.80 months vs. 3.40 months, p=0.45), or those who switched into non-gemcitabine-based regimen and those who continued or stopped gemcitabine-based chemotherapy (mPPS 3.40 months vs. 4.20 months, p=0.66). Potential difference in mPPS was observed between PR patients who added LRT after resistance and those who received systemic therapy only (mPPS 9.03 months vs. 3.08 months, p=0.057).

mPPS: median post-progression survival; ICI, immune checkpoint inhibitor; LRT, local regional therapy.


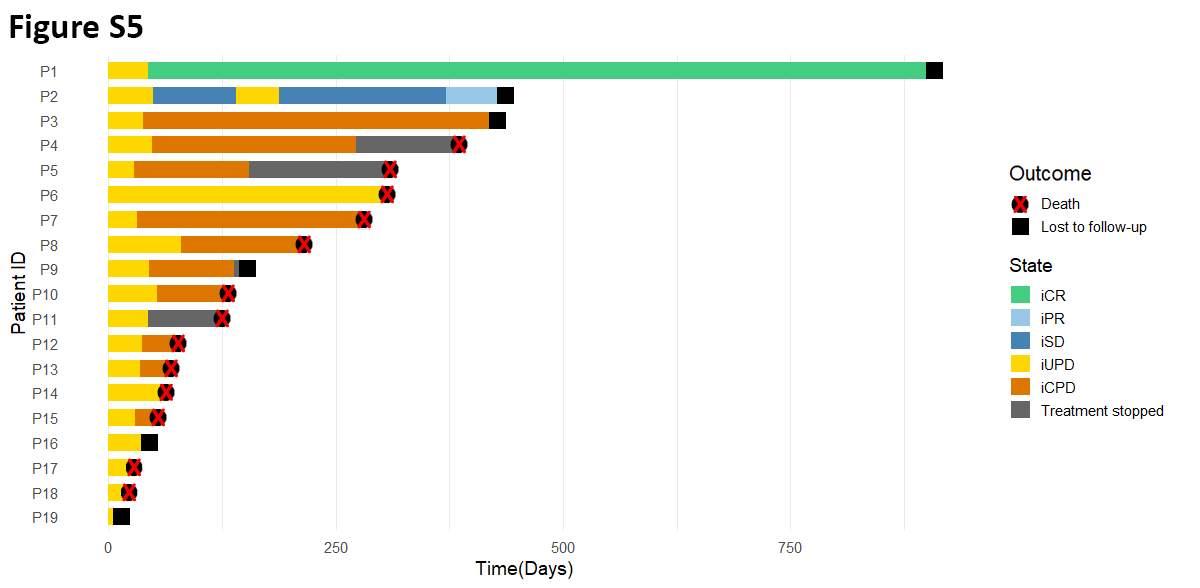
 Supplementary Fig. 5.

Swimmer plot of 19 patients who continued their initial treatment after progression. Among them, P1 and P2 were retrospectively determined as pseudoprogression according to iRECIST criteria.

“i” indicates immune responses assigned using iRECIST. RECIST, Response Evaluation Criteria in Solid Tumours; iUPD, unconfirmed progression; iCPD, confirmed progression; iCR, complete response; iPR, partial response; iSD, stable disease.
